# Behavioural responses to unexpected changes in reward quality

**DOI:** 10.1038/s41598-018-35056-5

**Published:** 2018-11-09

**Authors:** Stefanie Riemer, Hannah Thompson, Oliver H. P. Burman

**Affiliations:** 1 Division of Animal Welfare, VPH Institute, Vetsuisse Faculty, University of Bern, Längassstrasse 120, 3012 Bern, Switzerland; 2 Animal Behaviour, Cognition and Welfare Research Group, School of Life Sciences, University of Lincoln, Joseph Banks Laboratories, Green Lane, LN6 7DL UK

## Abstract

Successive negative contrast (SNC) effects are changes in anticipatory or consummatory behaviour when animals unexpectedly receive a lower value reward than they have received previously. SNC effects are often assumed to reflect frustration and appear to be influenced by background affective state. However, alternative explanations of SNC, such as the functional-search hypothesis, do not necessarily imply an aversive affective state. We tested 18 dogs in a SNC paradigm using a patch foraging task. Dogs were tested in two conditions, once with the low value reward in all of five trials (unshifted) and once when reward value was altered between high and low (shifted). Following a reward downshift, subjects showed a SNC effect by switching significantly more often between patches compared to the unshifted condition. However, approach latency, foraging time and quantity consumed did not differ between conditions, suggesting non-affective functional search behaviour rather than frustration. There was no relationship between strength of SNC and anxiety-related behaviours as measured in a novel object test and a personality questionnaire (C-BARQ). However, associations with the C-BARQ scores for Trainability and Stranger directed aggression suggest a possible link with behavioural flexibility and coping style. While reward quality clearly affects incentive motivation, the relationship between SNC, frustration and background affective state requires further exploration.

## Introduction

Incentive contrast effects – or animals’ responses to unexpected changes in reward value – have been of interest to researchers for many decades, starting with Elliott’s (1928)^1^ observation that rats reduced their running speed and entered more blind alleys in a maze when the reward was downshifted from the preferred bran mash to less valued sunflower seeds. Importantly, rats did not only run more slowly in the seed condition than in the bran mash condition, but they reduced their running speed below that of an unshifted control group that received sunflower seeds during all trials^1^. A classic study by Crespi^2^, using a runway task, similarly demonstrated that rats’ running speed was significantly reduced following a reduction in reward quantity compared to unshifted controls. Such a change in behavioural responses following an unexpected downshift in quality and/or quantity of reward, relative to an unshifted control group that has only ever received the lower value reward, is referred to as a “successive negative contrast” (SNC) effect^3^.

These behavioural changes are often interpreted as reflecting an aversive emotional state, induced by the discrepancy between the expected and actual level of reward^3–7^, but see^8,9^. According to Amsel’s^10^ frustration theory, the consequences of “surprising nonreward (SN)”, are as follows: Firstly, SN leads to an aversive emotional state of primary frustration. Secondly, the stimuli associated with the frustrating event will trigger secondary or anticipatory frustration, leading to their avoidance in the future. Thirdly, animals become tolerant to SN through counterconditioning (pairing of secondary frustration with reward), which ultimately increases behavioural persistence when faced with SN (reviewed by Papini^11^).

In the case of a SNC effect, *primary frustration* may manifest as behavioural changes when the subject is in direct contact with the reward^12^, such as an increase in search behaviour and exploration^3,13,14^, activity^13,15,16^, rearing^15^, and, most frequently, a reduction in consumption of liquid or solid food rewards (e.g.^14,17,18^). *Secondary frustration* may be reflected in behavioural changes preceding the acquisition of reward, such as slower running speeds in the examples cited above and lowered effectiveness in operant responding^16^.

However, alternative explanations of SNC exist which do not necessarily imply an accompanying aversive affective state, and studies that employed more naturalistic tasks (i.e. those more closely allied to ecologically relevant behaviours) than the classical studies of SNC in the laboratory, found that the subjects’ behaviour only partially supported frustration theory. In studies on rats^9^ and starlings^13^, food was available in several locations (a four-arm maze^9^ or different feeding stations^13^, respectively), thus allowing testing of alternative hypotheses such as the functional-search hypothesis, which states that a motivational change occurs from consumption to exploration when animals receive a lower value reward than expected^9,13^. As would be predicted by Amsel’s frustration theory^19^, subjects in both studies exhibited a SNC effect in consumption, as well as increasing activity and exploration after a reward downshift^13^. However, the frustration hypothesis would additionally predict that, when several options are available, animals should avoid the locations in which they have experienced the downshift (and thus the associated frustration) due to the aversive emotional state previously experienced in this location – but neither study found this to be the case. Instead, data were in line with the functional-search hypothesis^9^ and with ecological models of patch exploitation^20^, which predict that foragers should switch from a patch to sampling the environment when yield is lower than expected^13^.

Thus, results from previous studies are inconclusive. Moreover, although SNC effects have now been demonstrated in a number of mammalian species (e.g. rats^3,9^, mice^21^, sheep^16,22^, fallow deer^23^, and two opossum species^7,24^), a bird species (starlings^13^) and insects (honey bees; bumble bees^8,25^), most of our knowledge on the SNC effect and the factors influencing it is derived from studies on rats. However, as pointed out by Freidin *et al*.^13^, differences in energetic requirements, metabolism and perceptual systems between species makes an extrapolation of these results difficult.

Recently, interest in SNC has been renewed by suggestions that the strength of contrast may serve as an indicator of background emotional state and thus constitute an indirect measure of animal welfare^26,27^. There is some evidence that humans and non-human animals respond more strongly to negative events when in a negative emotional state (e.g.^26,28,29^) and in line with this interpretation, Burman *et al*.^26^ found that rats from barren housing took longer to recover following a reward downshift than rats from enriched housing. In contrast, Mitchell *et al*.^27^ found a paradoxical attenuation in SNC effect for rats housed in barren compared to enriched cages, but attributed this to a rebound in positive affect due to the comparatively enriching effects of daily testing. The existence of an emotional component to SNC effects is furthermore supported by differential performance in SNC tests of rat strains selected for high or low anxiety (e.g.^30–33^), as measured, for instance, by responses to novelty and exploration of novel objects^34–36^.

However, few studies have investigated the validity of SNC effects outside of highly standardised laboratory conditions, a precondition if they are to serve as a welfare indicator in farm and pet animals. Thus, we chose to investigate the external validity of the SNC paradigm and the role of SNC in influencing affective state in the domestic dog, *Canis familiaris*, a species in which a consummatory SNC effect in a modified SNC instrumental task has previously been demonstrated^4^, although see^37^. As well as being an increasingly popular animal model for studying cognition (e.g.^38–40^), the question of how dogs react to unexpected changes in reward quality is additionally of practical interest, since positive reinforcement training using food rewards is frequently used in this species (e.g.^41^). Yet, few studies have explored how to use this reward type most effectively (but see^42,43^) or the potential emotional effects of devaluation or omission of rewards (see^4,44–46^ for related research); thus, increasing our understanding in this area could potentially have a significant impact given the large numbers of dogs kept as companion animals (e.g. c. 10.5 million dogs in the UK^47^).

The aims of the current study were therefore (1) to investigate the occurrence of SNC in dogs using a naturalistic novel foraging task and (2) to test the prediction that strength of SNC effect is related to emotional reactivity.

Eighteen privately owned pet dogs (*Canis familiaris*) were tested in a foraging task, in which food was provided in four ‘activity boards’, laid out in a cross formation (see Methods section). Boards were filled with 18 pieces of either a high value food or a low value food (as confirmed in a preliminary preference test), which required some effort for the dogs to extract (see Methods). In the unshifted condition, dogs received the low value food in all of five trials, whereas in the shifted condition, dogs received the high value food in trials 1 and 2 (pre-shift phase), followed by a downshift to the lower value food in trials 3 and 4 (post-shift phase). In trial 5 (re-shift phase) they received the high value food again. The study used a within-subjects design so that all dogs experienced both the shifted and the unshifted condition, with order of presentation counterbalanced between subjects.

Additionally, given associations with the strength of the successive negative contrast effect and anxiety in rats^31,32,48,49^, dogs’ reactions towards a novel (moving and noise-producing) object were observed in order to obtain a measure of anxiety. As an additional validated measure of canine personality, dog owners filled in the C-BARQ (Canine Behavioral Assessment and Research Questionnaire)^50^.

## Results

### Latency to engage with the first board

**Figure 1:**
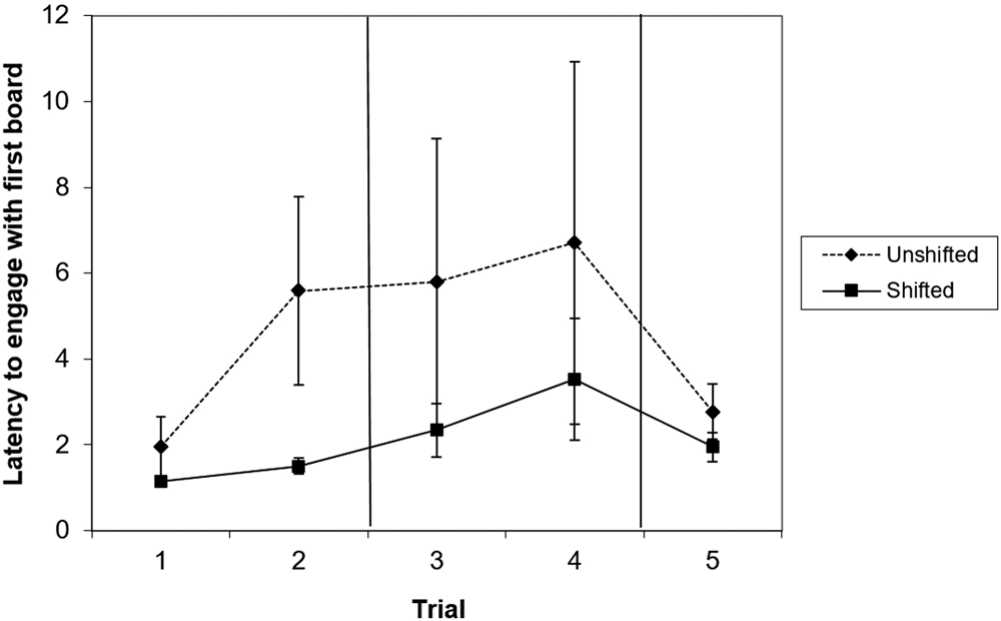
Mean and standard error of dogs’ latency (seconds) to engage with the first board in trials 1–2 (preshift), 3–4 (post-shift) and 5 (re-shift) of the unshifted and the shifted condition. N = 18.

This measure was analysed only for the second pre-shift trial and the second post-shift trial (i.e. trials 2 and 4), since dogs could not yet have an expectation of the reward on offer in the respective first trials of each shift phase. Although approach latency overall appears to be longer in the unshifted condition compared to the shifted condition (Fig. 1), this was not significant in either Trial 2 (Wilcoxon Z = 1.65, p = 0.09) or Trial 4 (post-shift: Wilcoxon Z = 0.85, p = 0.39).

### Time engaged with boards

**Figure 2:**
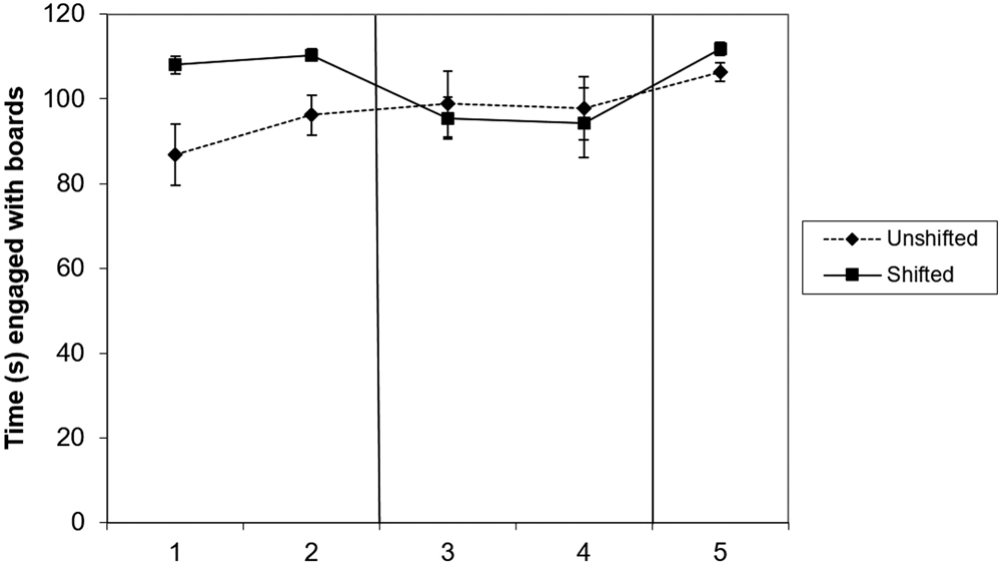
Mean and standard error of time (seconds) spent interacting with the boards in trials trials 1–2 (preshift), 3–4 (post-shift) and 5 (re-shift) of the unshifted and the shifted condition. N = 18.

Dogs spent significantly more time engaging with the boards when they contained the high value food than when they were filled with the low value food in both pre-shift and re-shift trials (Table 1). In contrast, during post-shift trials, when all boards were filled with the low value food, there was no effect of shift condition on time spent engaged with the boards (Fig. 2; Table 1).

**Table 1:** Results of Linear Mixed Models.

Dependent variable	Phase	Predictor(s)	Estimate	Std. Error	CI −95%	CI + 95%	numDF	denDF	F	p
Time engaged with boards	Preshift	Treatment	−17.89	3.85	−26	−10.2	1	53	21.6	<0.0001*
	Postshift	Treatment	1.12	5.2	−9.3	11.56	1	53	0.46	0.8301
	Reshift	Treatment	−6.91	2.01	−11	−2.02	1	17	11.8	0.0032*
Number of pieces consumed	Preshift	Treatment	−4.67	2.28	−9.3	−0.08	1	53	4.16	0.0464
	Postshift	Treatment	0.3	1.99	−4.3	3.7	1	53	0.02	0.8791
	Reshift	Treatment	3.11	−0.49	−10	6.03	1	17	0.25	0.6238
Frequency of switches	Preshift	Treatment	1.4	0.5	0.18	2.43	1	53	5.44	0.0234*
	Postshift	Treatment	−10.22	4.32	−19	−1.53	1	51	5.57	0.0221*
		Trial	−1.88	0.86	−3.6	−0.15	1	51	4.76	0.0338
		Treatment:Trial	2.55	1.22	0.1	5.01	1	51	4.36	0.0419
	Reshift	Treatment	1.5	0.74	0.23	3.33	1	17	4.08	0.0593

*marks results that are significant after correction for multiple testing, using False Discovery Rate Control. N = 18.

### Total number of pieces eaten

**Figure 3:**
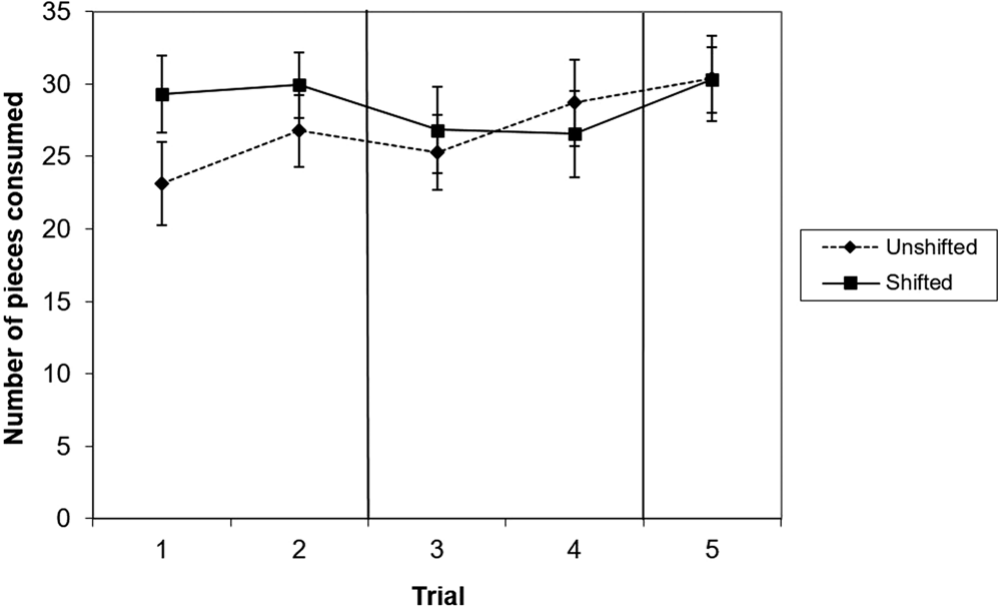
Mean and standard error of number of pieces eaten in trials 1–2 (preshift), 3–4 (post-shift) and 5 (re-shift) of the unshifted and the shifted condition. N = 18.

During pre-shift trials, dogs consumed a greater number of pieces in the shifted condition than in the unshifted condition, indicating a preference for the high value food, although this effect was no longer significant following correction for multiple testing (Table 1, Fig. 3). There was no effect of shift condition in post-shift trials or re-shift trials (Table 1, Fig. 3).

### Number of switches between boards

**Figure 4:**
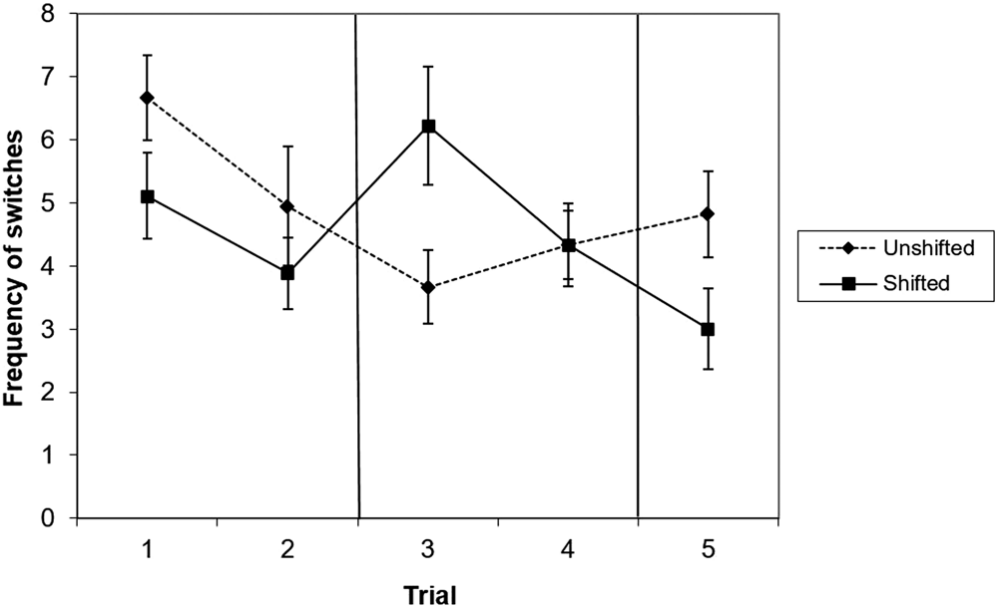
Mean and standard error of number of switches in trials 1–2 (preshift), 3–4 (post-shift) and 5 (re-shift) of the unshifted and the shifted condition. N = 18.

In the pre-shift trials, dogs performed significantly more switches between boards in the unshifted condition than in the shifted condition (Table 1, Fig. 4). During the post-shift phase, a successive negative contrast effect was observed in number of switches: after being confronted with a reward downshift, dogs significantly increased the number of switches compared to the unshifted condition. While these results appears to be driven by the first post-shift trial only, the effects of trial and the treatment:trial interaction were no longer significant following correction for multiple testing. During re-shift, there was again a tendency towards a higher number of switches in the unshifted condition.

None of the results were affected by treatment order, which was initially included as a random factor in the models, but was subsequently removed in all models due to the lack of a significant influence.

### SNC score calculation

A SNC score was calculated for each dog based on the difference in the mean number of post-shift switches between their shifted and unshifted conditions (see Supplementary Table S12), with higher scores indicating a greater difference, and thus a stronger SNC effect.

### SNC score and personality scores

**Figure 5:**
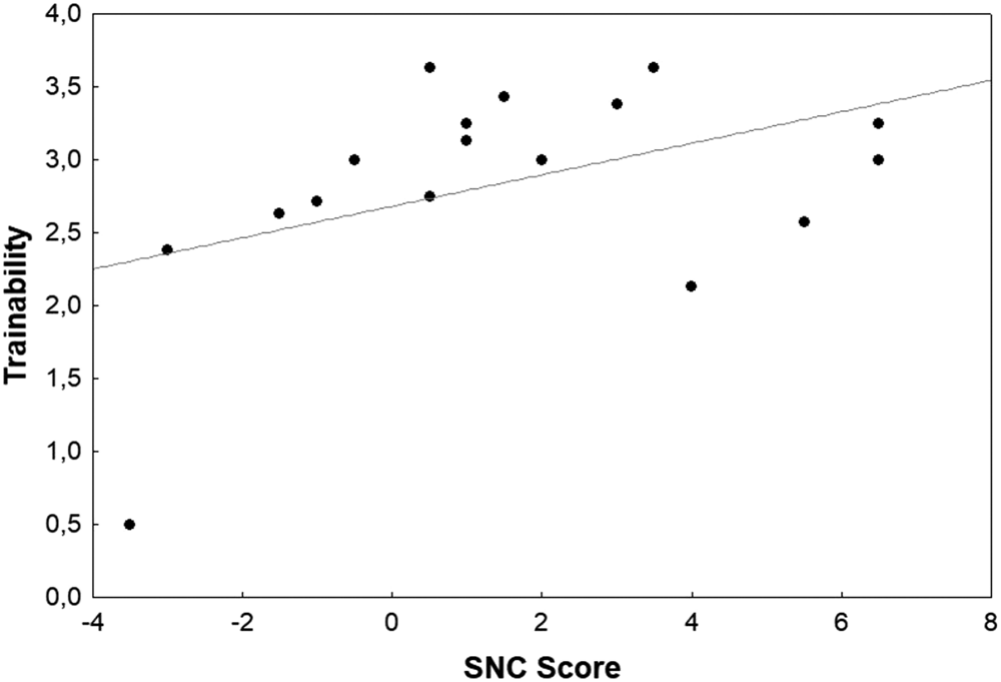
Trainability plotted against the SNC score. N = 18.

**Figure 6:**
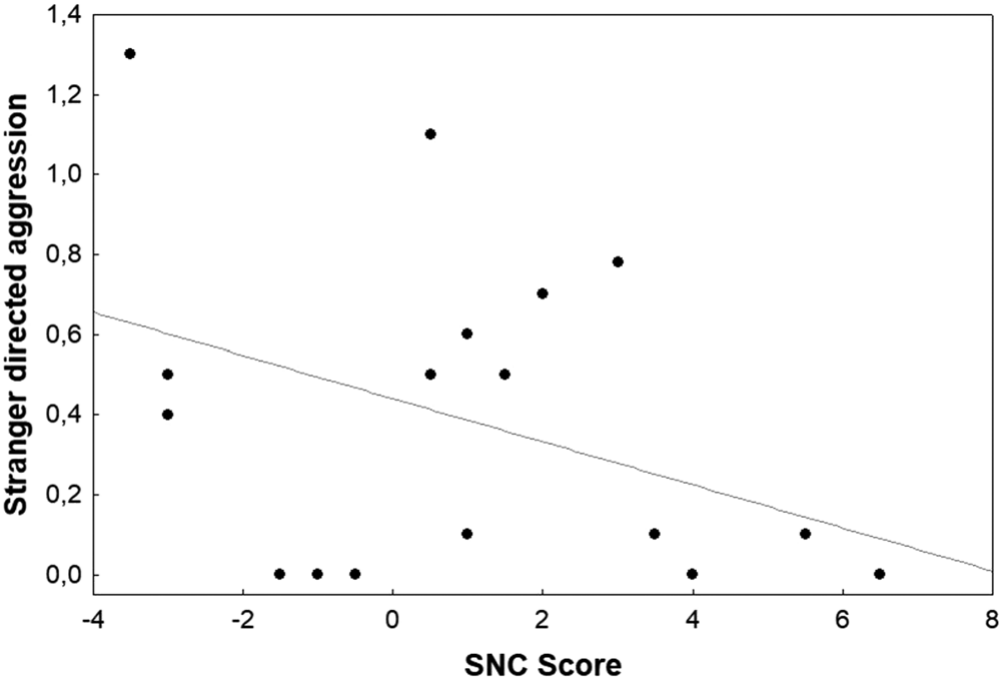
Stranger directed aggression plotted against the SNC score. N = 18.

Given the hypothesised association of strength of SNC with personality, the SNC score was related to C-BARQ scores using linear models. The best model according to Akaike Information Criterion explained 20.19% of the variance and included the predictors Trainability, Stranger directed aggression and excitability (adjusted R^2^ = 0.2019, F_3,13_ = 2.349, p = 0.12). The results indicated a significant positive relationship between SNC score and Trainability (β = 0.540, p = 0.048; Fig. 5), whereas the SNC score was significantly negatively associated with Stranger directed aggression (β = −0.722, p = 0.036; Fig. 6). The effect for Excitability, although included in the best model, was not significant (β = 0.458, p = 0.138).

### Novel object test, ‘Nonsocial fear’ and SNC score

The Categorical Principal Components Analysis on the startle score and the proximity score from the novel object test yielded a single principal component that accounted for 90.55% of the variance (Cronbach’s Alpha 0.90, Eigenvalue 1.81), with higher values on this ‘novel object score’ indicating bolder/less anxious behaviour. Both the startle score and the proximity score had loadings of 0.95 on this component. However, although an association of the Novel object score with the C-BARQ score ‘Nonsocial Fear’ was expected, this was not the case (Spearman Rho = −0.18, N = 18, p = 0.46), and neither was the novel object score related to the SNC score (Spearman Rho = −0.04, N = 18, p = 0.88).

## Discussion

Using a within-subjects design, we performed a SNC experiment using a patch foraging task in which pet dogs could extract food rewards of either high or low value from four simultaneously available boards. The dogs’ sensitivity to differences in reward quality was confirmed by both a preliminary preference test and by behaviour during pre-shift trials: subjects spent more time interacting with the boards and showed less switching between boards when they were filled with the high value food than when they were filled with the low value food. Additionally, consumption tended to be greater for the former.

After experiencing a reward downshift, dogs showed a temporary successive negative contrast effect by increasing the number of switches between boards above the level shown in the unshifted condition. Unlike in other species tested with multiple feeding stations (starlings^13^, rats^9^, bumblebees^25^), consumption after the downshift did not fall below that in the unshifted condition. Rather, time spent interacting with the boards and consumption seemed to be directly related to absolute reward quality, being generally higher for the high quality food than for the low quality food (replicating findings from other studies on SNC in dogs^37,42^, but see^4^). Furthermore, despite the greater number of switches following the downshift, time spent engaged with the boards in the post-shift phase did not differ between the shifted and the unshifted condition. If we consider that consummatory SNC effects occur because downshifted individuals spend more time exploring (to find the ‘missing’ expected high quality food), leaving less time for consumption, the lack of difference in post-shift consumption and thus the divergent results from^9,13^ and^25^ could potentially be explained by the close proximity between the boards, and thus minimal time required for switching. However, evidence from Freidin *et al*.^13^ indicates that temporal constraints do not account for the observed decrement in consumption.

After Amsel^10^, behavioural changes when in contact with the reduced quality reward, such as a reduction in consumption and increased exploration, are indicative of primary frustration, while secondary frustration subsequently manifests as behavioural changes prior to acquisition of the reward, such as slower running speeds, reduced operant behaviour, or avoidance of the location of the downshift^10,16^. However, whether switching really reflects an aversive emotional response has been questioned. Alternatively, it can be viewed as a non-affective functional response with a motivational change to higher exploration^9,13,25^. For instance, the functional-search hypothesis postulates that SNC encourages foragers to visit alternative food sources, thus facilitating economical decisions without a concomitant emotional response^9,13,25^. In a related vein, Flaherty’s^3^ multi-stage hypothesis postulates that downshifted animals quickly detect the discrepancy in reward (resulting in the initial decrease in consumption and increased search behaviour), without necessarily experiencing an aversive state. However, stress occurs when the animal is unable to recover the original, preferred reward during subsequent search behaviour; i.e., stress is a delayed consequence of reward reduction^51^.

In the present study, there was no indication of such a stress response (or secondary/anticipatory frustration after Amsel^10^) upon being unable to recover the expected high-quality reward, since latency to engage with the first board did not increase in the second post-shift trial. Considering that frustration may not kick in immediately^3^, perhaps not enough trials were conducted for these delayed consequences to become apparent, or the two-minute access to the downshifted reward was not sufficiently long for the development of conditioned frustration effects. The number of pre-shift trials was also limited to just two exposures in order to shorten the procedure and make it more applicable as a potential welfare measure. This could have led to the relatively weak SNC effect, and it is also possible that the observed differences were influenced by both conditions (shifted/unshifted) having insufficient time to achieve asymptotic levels. Although pre-shift exposure only needs to be sufficiently long to allow an expectation to be formed (e.g.^52^), and change in performance reflected our predictions for the different stages (pre-shift/post-shift/re-shift), refinement of this task in the future could include providing additional preshift and post-shift trials to strengthen the SNC effect and/or allow sufficient time for frustration effects to develop.

Thus, apart from the increased number of switches, our subjects showed no evidence of either primary or secondary frustration. Nonetheless, we cannot rule out that our measures were not suitable to detect frustration in this context. There is currently a lack of studies on how the emotion of frustration might be expressed in dogs. Although one study included frequencies of yawning, lip-licking, stretching, scratching/grooming itself, and shaking into a composite score for frustration in dogs, these behavioural signs were not actually validated as expressing frustration^53^. Two other studies, using a social context in which food was withheld from the dogs, reported increases in withdrawal from the human, side orientation to the location of the human, lying down, ambulation, sniffing, turning in circles, biting/chewing the experimenter’s hand (which contained food) and vocalizations^44,46^. Neither of these two studies identified putative “stress signs” such as lip-licking or yawning^54^ as indicating frustration. As the dogs’ faces were directed downwards for the majority of time, a coding of such subtle stress signals was not feasible in the current study and we had to rely on more overt behavioural changes. However, studies indicate that emotional changes may not necessarily manifest behaviourally^55^. For example in rats, ultrasonic vocalisation but not behavioural parameters changed when exposed to a reduction in reward in some contexts^55,56^, and the opposite was found in other contexts^56^.

Generally, it appears from several recent studies that while dogs are sensitive to reward quality, they react less strongly to surprising reward downshifts than other species. For instance, in Riemer *et al*.^37,42^, operant responding was adapted to reward quality, but dogs showed no exaggerated behavioural responses (SNC) following an unexpected reduction in reward value (but see^4^). Likewise, no direct behavioural effect of rewards of varying quality were observed in two studies on inequity aversion in dogs^57,58^. Whereas dogs that received no reward refused to cooperate sooner with the experimenter (by giving the paw) in the presence of a rewarded conspecific than in a control condition (no reward, no partner), neither rate of giving the paw nor stress behaviours differed between conditions where both dogs were rewarded with either equal or unequal rewards^57,58^. Nonetheless, despite the lack of overt behavioural responses during the test, Brucks *et al*.^58^ did find evidence of behavioural changes outlasting the actual testing situation, with dogs that had received qualitatively different rewards showing reduced cooperation (as measured by duration of food sharing) in a subsequent tolerance test^58^. Thus, it is possible that the dogs were aware of the downshift but were lacking the inhibitory control to express any negative emotions more overtly^58^, such as by refusing the operant action or the lower value food, and the same may have been the case also in Riemer *et al*.^37,42^ and the current study.

It is a possibility that dogs’ experiences with varied reward in everyday life make them less susceptible to SNC effects (c.f.^3^). That is, dogs are often required to perform actions that are only rewarded during some of the time, and with rewards of variable qualities, thus experiencing counter-conditioning to the aversive effects of such reward schedules (see^59^). Given that dogs appear to habituate to a given reward type even when it is of high value, varying reward types is even suggested to be an effective way of enhancing performance motivation over a longer time period (c.f.^43^). Finally, it is suggested that individuals with a more positive background affective state are less susceptible to SNC^26^. As pet dogs, all our subjects came from highly enriched conditions, potentially explaining the low susceptibility to mildly frustrating events compared to most subjects in laboratory studies.

To gather additional evidence of a possible emotional influence on SNC, we related strength of SNC to dogs’ reactions towards a novel object. This was carried out on the basis that in rats, individuals of highly anxious lines not only showed less exploratory behaviour and more pronounced physiological reactions to stressors, but they also showed a more pronounced SNC effect (e.g.^30–33^). However, in the current study, no relationship between SNC score and novel object score was found. This could indicate, firstly, that fearfulness towards novel objects is unrelated to aversive emotions following a reward downshift in dogs, secondly, that the search behaviour displayed by the dogs following the downshift was not associated with an aversive emotional state, or thirdly that either or both of the tests performed do not allow inferences about the dogs’ (background) emotional state. Even when using the C-BARQ, measures of fearfulness/anxiety did not correlate with the SNC score; instead the SNC score was positively associated with Trainability and negatively with Stranger directed aggression.

Thus, the behaviour observed in the SNC test may not reflect an affect-related response, but instead, the change in number of switches following a downshift could be indicative of behavioural flexibility. It is conceivable that the behaviourally more flexible individuals were more sensitive in perceiving the change in reward value and reacting to it (c.f.^60^), and this behavioural flexibility could translate to improved learning and thus higher trainability in everyday life. Moreover, stranger directed aggression was negatively associated with the SNC score. Behavioural flexibility is suggested as an important factor underlying behavioural consistency across situations in coping styles^61^, and a number of studies indicate that low behavioural flexibility and a more proactive coping style are linked^61^. For example in piglets, individuals that showed higher resistance when restrained for one minute in the “back test” were less successful in reversal learning than low-resisting individuals^62^. Individuals with more proactive coping styles typically exhibit low flexibility and thus “rigid, routine-like behavioural tendencies in operant conditioning paradigms” compared to the more flexible individuals with a reactive coping style^61^. Proactive coping styles (low flexibility) are associated with higher levels of offensive behaviour^61^, and this might be reflected in the greater persistence in the SNC task observed in subjects scoring higher on Stranger directed aggression. Note, however, that not all types of aggressive behaviour were associated with SNC score, as Dog-directed aggression was not included in the best model according to AIC. Thus, more data are needed to explore a possible association between individual behavioural flexibility, coping style and sensitivity to reward change.

## Conclusions

To conclude, we found that dogs in the current study showed a SNC effect in their search behaviour when tested using a novel naturalistic foraging task, but this was not reflected in other measures of SNC performance such as total consumption. While the strength of the SNC effect was unrelated to measures of fear/anxiety (as determined by C-BARQ scores and responses to a novel object), associations with the C-BARQ scores for Trainability and Stranger directed aggression suggest a possible link between sensitivity to reward reduction, behavioural flexibility and coping style. In general, dogs appear to be relatively insensitive to unexpected changes in reward quality compared to other taxa, possibly because their experiences with varied reward in everyday life make them less susceptible to SNC effects (c.f.^3,10^). Whether or not SNC necessarily indicates a negative affective state remains inconclusive and caution should be taken when associating behavioural changes in response to unexpected switches in reward value with a concomitant alteration of affective state. While reward quality clearly affects incentive motivation in dogs, the relationship between SNC, frustration and background affective state requires further exploration.

## Methods

This work followed the Association for the Study of Animal Behaviour (ASAB) guidelines for the use of animals in research and was approved locally by the Research Ethics Committee of the School of Life Sciences at the University of Lincoln. Dog owners gave written consent for their dogs to be included in the study.

### Subjects

Subjects were 18 privately owned pet dogs (8 female, 7 neutered; 10 male, 6 neutered) of various breeds, ranging in age from 10 to 144 months (mean 39.4 months; Table S1). The dogs were recruited via the University of Lincoln’s PetsCanDo data base of volunteer dog owners. Only dogs in good health that had no history of resource guarding or serious human-directed aggression were included. Dogs were not food-deprived for the study except that owners were asked not to feed them for four hours prior to the experiment.

### Food rewards

The two types of food reward were chosen to be of different hedonic value to the dogs yet of similar ease to extract from the ‘activity board’ (see below), cut into pieces of the same size. Food type 1 was “Arden Grange Adult dog food with Chicken™” (crude protein 25%, fat 15%). Food type 2 were “Pets at Home Meaty Sticks with Beef and Game” (crude protein 40%, fat 20%). In order to confirm the relative value of the two food types, dogs were observed in a food preference test, in which time spent investigating the inaccessible food was used as a proxy for preference (see Thompson *et al*.^63^ for details). Reliability between the first and second author in coding the food preference test has previously been demonstrated^42^. The preference test confirmed that Food type 2 was significantly preferred to Food type 1 (t-test: t = 6.31, DF = 17, p < 0.001). Except for one dog that failed to interact with either food type, all dogs spent more time investigating Food type 2 (mean 24.11 ± 2.32 seconds) than Food type 1 (mean 7.61 ± 2.32 SEM). Food type 2 was therefore designated as the ‘high value’ reward and Food type 1 as the ‘low value’ reward. Because all the dogs consumed all food when it was accessible, all dogs (N = 18) were included in the SNC test.

### SNC experiment

**Figure 7:**
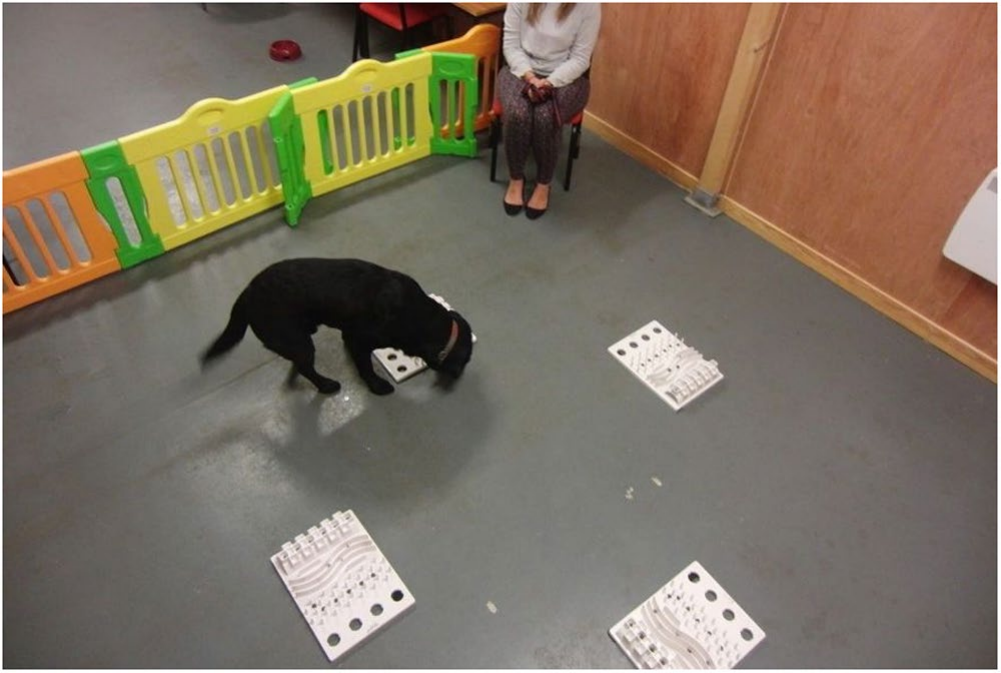
Setup of the test area showing the position of the four activity boards and the chair of the owner. The experimenter’s chair was located in the corner opposite that of the owner (not visible on the photo).

Testing was conducted in a room at the University of Lincoln. The testing area (3.5 × 3.5 m) was enclosed by solid walls on three sides and by a 70 cm high plastic barrier on the fourth side. We used a foraging task, in which food was provided in ‘activity boards’ at four different locations (Fig. 7). Activity boards (“Trixie Cat Activity Fun Boards”) are 30 × 40 cm plastic toys originally designed for cats, featuring several pegs, slots and dents from which food treats can be extracted, so that some effort is required to obtain the food. The removable plastic bowls from the original boards were removed for safety reasons.

#### Familiarisation

To familiarise the dogs with the boards prior to testing, they were first presented with a single activity board in a room adjacent to the room where SNC testing was conducted. This board was filled with 18 pieces of the same food type as the dogs were going to receive in the first trial of the SNC test (low value food if in the unshifted condition, high value food if in the shifted condition, see later). The dog was given off-lead access to the activity board for a maximum of ten minutes. Initially, the owner or experimenter provided encouragement or even helped the dog to obtain treats when needed, but the aim was to encourage independent problem solving. When the dog had consumed half the food in the activity board, the experimenter and the owner ignored the dog for the rest of the trial. The familiarisation trial ended when the dog had obtained all treats or after 10 minutes. During this time period, all dogs consumed at least 12 food pieces. Subsequently, the dog was given a break of five minutes.

#### Test procedure

For testing, four activity boards were laid out in a cross formation in the centre of the testing area, as shown in Fig. 1. Five trials were performed. During each of the five trials, all four activity boards were filled with 18 pieces of the same food type, depending on the condition. In the unshifted condition, the boards were filled with the low value food (according to the initial preference test, hereafter referred to as “low value reward”) in all five trials [*low*, *low*, *low*, *low*, *low*]. In the shifted condition, the boards were filled with the high value food (according to the preference test, hereafter referred to as “high value reward”) for two trials (pre-shift phase), followed by a downshift to the low value food for two trials (post-shift phase). In the final (re-shift) trial the high value food was provided again [*high*, *high*, *low*, *low*, *high*]. This re-shift to the high-value reward was introduced to control for satiation effects, c.f. Bentosela *et al*.^4^. The study used a within-subjects design (c.f.^37,42^) to account for individual variation and increase statistical power (e.g. Keren & Lewis^64^). Each dog was tested on two occasions, approximately one week apart. The order of presentation (shifted condition in one week, unshifted condition in the other) was counterbalanced between subjects to avoid systematic bias. The total number of trials in each condition (five) was deliberately limited in order to shorten the duration of testing whilst providing sufficient time (due to long trial durations) for an expectation of reward to be formed, thus maintaining subject motivation and making the task more applicable as a potential welfare measure.

During each trial, following preparation of the four boards, the experimenter sat in her designated chair inside the test area. The owner then entered with the dog, sat down on his/her chair in the opposite corner inside the test arena and released the dog upon instruction from the experimenter. From the moment of release, the dog had two minutes access to the activity boards while all people ignored the dog completely. The experimenter indicated to the owner when this time was over, and the owner lured the dog away from the activity boards. The experimenter, owner and dog then exited the test area together. Following a 10 minute inter-trial interval outside of the test room, the next trial commenced.

All trials were video-taped. From the videos, latency to engage with the first board after release, duration of time engaged with boards, and number of switches between boards during each two-minute trial were analysed in Solomon Coder (© András Péter). Reliability with a second coder was analysed for twenty videos and was excellent (latency to engage with the first board: Cronbach’s alpha = 1.0; duration of time engaged with boards: Cronbach’s alpha = 0.997; number of switches between boards; Cronbach’s alpha = 0.996). We recorded the number of pieces consumed directly after each trial. This use of multiple performance-related measures was intended to allow additional insight into the affective motivation of the dogs. In pilot studies, as well as the main study, ad libitum observations indicated no clear postural changes between conditions nor differences in vocalisations. All dogs spent the majority of time with their head lowered as they were foraging. As a result, neither head position nor ear position were suitable to infer possible stress, because, for floppy-eared dogs, their ears hung down owing to the lowered head position. The mouth was also often not visible in the videos while dogs were foraging, and, additionally, measuring lip licking as a sign of stress in a feeding context could have been misleading, as it could also be performed due to feeding. Such behaviours were therefore not recorded in the current study.

### Novel object test

We included a novel object test in order to identify whether differences in putative affective state, as measured by the SNC test, were reflected in anxiety-related behaviour. The novel object was a 30 cm high battery-operated “Singing and Dancing Musical Christmas Tree”, which played a song and performed dancing movements, placed at the back of the wall of a small test room (3.7 × 3.7 m). Dogs were released into the test room when the toy was already turned on and the door was left open so that dogs could remove themselves from the situation if they wished to do so. The dogs’ initial startle reaction was coded as detailed in Table 2, and time spent looking at and within 1 m of the novel object were coded for 20 seconds. A proximity score – as a measure of anxiety – was calculated by dividing the time dogs spent within 1 m of the object by the duration of looking at the object, to control for dogs that lost interest quickly and therefore spent little time in its vicinity. These measures were selected to best encompass the range of anxiety-like behaviours displayed by the different dogs.

**Table 2:** Scoring of startle response upon exposure to the novel object (within the first three seconds).

0	moves away from the novel object
1	passive (no reaction in the first 3 s, only looking)
2	walks around to look at toy from a different angle but does not move closer than 1 m within the first 3 s
3	approaches hesitatingly to within 1 m
4	approaches toy immediately to within 1 m

### Owner questionnaire

As a measure of dogs’ personality, all dog owners filled in the Canine Behavioral Assessment and Research Questionnaire (C-BARQ), a validated questionnaire with a focus on behavioural problems in dogs yielding scores for thirteen traits, Stranger directed aggression, Owner directed aggression, Dog directed aggression, Familiar dog aggression, Dog directed fear, Nonsocial fear, Stranger directed fear, Separation related problems, Trainability, Chasing, Touch sensitivity, Excitability, and Energy^50^.

### Analysis

Statistical analysis was performed in R 3.3.3 (R-project.org).

#### SNC Task

For each phase (pre-shift, post-shift and re-shift), linear mixed effect models (function lme, R package nlme^65^) were calculated to assess effects of Treatment, trial, and the interaction between these predictors on behavioural variables, with dog ID nested in Treatment order included as a random factor. Models were reduced stepwise (backwards selection) if trial or the interaction between trial and reward shift group were non-significant. Lastly, the final reduced model was compared with and without the inclusion of Treatment order as a random factor. Residuals of the models were assessed for meeting the assumptions of parametric testing, and these were adequate for all models. For each dependent variable, False Discovery Rate (FDR) control correction^66^ was applied to correct for multiple testing (i.e. separate analyses for pre-shift, post-shift and re-shift phases).

For approach latency, only the second and fourth trial were analysed, since in the first pre-shift trial and the first post-shift trial dogs could not yet know what to expect. Since all but one of these trials were not normally distributed, Wilcoxon signed rank tests were used to compare dogs’ approach latency between the shifted and unshifted conditions in trials 2 and 4, respectively.

#### Reaction to novelty

The startle score and the proportion of time dogs spent within 1 m of the object while looking at it were subjected to a categorical principal components analysis (CATPCA^67,68^). The relationship between the novel object principal component and the “Nonsocial fear” score from the C-BARQ was assessed via Spearman rank correlation test.

#### Relationship of C-BARQ scores and novel object test score to SNC score

Linear models (function lm in R) were calculated to assess whether the SNC score was related to the C-BARQ scores Stranger directed aggression, Dog directed aggression, Dog directed fear, Nonsocial fear, Separation related problems, Trainability, Chasing, Touch sensitivity, Excitability, and Energy. Due to low variability in some of the scores relating to fear and aggression (i.e. ten or more subjects scoring zero for Owner directed aggression, Familiar dog aggression, and Stranger directed fear), these were not included in the models.

Given the large number of predictors, Akaike’s Information Criterion (AIC) was used to determine the model with the best fit (package MASS in R, function step, backwards selection, Type III sums of squares). Independence, normality, and homoscedasticity of the model residuals justified the modelling approach.

Data for the novel object score were not normally distributed, and therefore a Spearman rank correlation test was used to correlate this score with the SNC score.

## Electronic supplementary material

LaTeX Supplementary File

## Data Availability

The datasets generated and analysed during the current study are available from the corresponding author on reasonable request.
