# Supplementary material for: Behavioural responses to unexpected changes in reward quality
Source: Sci Rep. 2018 Nov 9;8:16652. doi: 10.1038/s41598-018-35056-5 (PMC6226435; doi:10.1038/s41598-018-35056-5)
Supplement: Supplementary file 1 — LaTeX Supplementary File [file 41598_2018_35056_MOESM1_ESM.docx]

Supplementary Data

# **Behavioural responses to unexpected changes in reward quality**

**Stefanie Riemer^1,2^, Hannah Thompson^2^, Oliver Burman^2,^***

**Electronic supplementary material**

**Table S1.** Demographic data.

| **Name** | **Sex** | **Neuter status** | **Age (months)** | **Breed** | **First condition** |
| --- | --- | --- | --- | --- | --- |
| Archie | male | intact | 36 | Beagle | Shifted |
| Archie | male | intact | 48 | Labradoodle | Unshifted |
| Pepper | male | neutered | 24 | Minature Schnauzer | Shifted |
| Toby | male | neutered | 72 | Standard Poodle | Shifted |
| Tutts | female | neutered | 24 | Mixed | Shifted |
| Zip | male | neutered | 34 | Border Collie | Unshifted |
| Poppy | female | neutered | 30 | Mixed | Unshifted |
| George | male | neutered | 24 | Labrador | Unshifted |
| Charlie | male | intact | 10 | Cockapoo | Unshifted |
| Hope | female | neutered | 69 | Labrador | Unshifted |
| Zoe | female | neutered | 144 | Flat coated Retriever | Unshifted |
| Kess | female | neutered | 11 | Labrador | Unshifted |
| Rue | male | neutered | 72 | Lurcher | Shifted |
| Juno | female | intact | 14 | Border Collie | Shifted |
| Penny | female | neutered | 26 | Greyhound | Unshifted |
| Jupiter | male | neutered | 20 | Labrador | Shifted |
| Buster | male | intact | 11 | Minature Schnauzer | Shifted |
| Jess | female | neutered | 40 | Mixed | Shifted |

**Table S2.** CBARQ scores of the subjects.

| **ID** | **Stranger directed aggression** | **Owner directed aggression** | **Dog directed aggression** | **Familiar dog aggression** | **Dog directed fear** | **Nonsocial fear** | **Stranger directed fear** | **Seperation related problems** | **Trainability** | **Chasing** | **Touch sensitivity** | **Excitability** | **Attachment attention seeking** | **Energy** |
| --- | --- | --- | --- | --- | --- | --- | --- | --- | --- | --- | --- | --- | --- | --- |
| **Archie beagle** | 1.3 | 0.25 | 2 | 1.25 | 0.91 | 2 | 3.5 | 1.67 | 0.5 | 1.88 | 0.5 | 2.33 | 2.67 | 1.33 |
| **Archie labradoodle** | 0 | 0 | 0.5 | 0 | 1.25 | 0.5 | 0 | 0.38 | 3 | 2.25 | 0 | 2.33 | 1.33 | 2 |
| **Buster** | 0 | 0 | 1 | 0 | 0 | 0 | 0 | 0 | 3 | 1.5 | 0 | 1 | 1 | 1 |
| **Charlie** | 0 | 0 | 0 | 0 | 0.5 | 2 | 0 | 0.75 | 3.25 | 2.25 | 0.5 | 1.83 | 2.6 | 3 |
| **George** | 0 | 0 | 0.75 | 0 | 1.25 | 0.5 | 0 | 0.13 | 2.63 | 1.75 | 0.25 | 3 | 1.67 | 3 |
| **Hope** | 0.5 | 0 | 1 | 0.5 | 0.5 | 1.5 | 0.75 | 0.13 | 3.43 | 3 | 0.75 | 2.17 | 2.17 | 0.5 |
| **Jess** | 1.1 | 0 | 1.75 |  | 1.33 | 1.67 | 1.5 | 0.38 | 3.63 | 1.75 | 3 | 2.83 | 2.33 | 2.5 |
| **Juno** | 0.5 | 0 | 1 | 0 | 2.5 | 1.33 | 0 | 1.13 | 2.38 | 1.5 | 0.75 | 2 | 2.33 | 1.5 |
| **Jupiter** | 0 | 0 | 0 | 0 | 0 | 0 | 0 | 0.13 | 2.71 | 1 | 0 | 0.83 | 1.67 | 1 |
| **Kess** | 0.1 | 0 | 0.25 | 0 | 0.75 | 0 | 0.25 | 0.5 | 3.63 | 2.75 | 0.75 | 1 | 2.5 | 2 |
| **Penny** | 0 | 0.38 | 0 | 0 | 0 | 0.67 | 0 | 0.13 | 2.13 | 0.5 | 0 | 1.67 | 1.5 | 0 |
| **Pepper** | 0.4 | 0 | 1.25 | 0 | 0.25 | 0.67 | 0 | 0.75 | 2.38 | 3.75 | 0.75 | 2 | 2.17 | 1.5 |
| **Poppy** | 0.6 | 0.25 | 0.5 |  | 1.25 | 1.17 | 0.75 | 0.13 | 3.13 | 2 | 1.33 | 3 | 2.67 | 3 |
| **Rue** | 0.1 | 0 | 2 |  | 2.33 | 1 | 0.5 | 0.38 | 2.57 | 3 | 0.25 | 2.4 | 2.2 | 2 |
| **Toby** | 0.5 | 0 | 0 | 0 | 1.5 | 1.33 | 0.5 | 0.71 | 2.75 | 2.5 | 0 | 2.83 | 1.33 | 3 |
| **Tutts** | 0.7 | 0.13 | 0 | 0.67 | 1 | 1 | 0 | 0 | 3 | 2.75 | 0 | 3 | 1.67 | 1.5 |
| **Zip** | 0.78 | 0.13 | 1 | 1 | 0 | 0.3 | 0.75 | 1.5 | 3.38 | 2.5 | 1.5 | 2.5 | 1.17 | 4 |
| **Zoe** | 0.1 | 0 | 0 | 0.5 | 0.25 | 1 | 0 | 0 | 3.25 | 2.75 | 0.67 | 1.17 | 1.5 | 0 |

**Table S3.** Time spent interacting with the two food types during the preliminary preference test.

| **Dog** | **High value** | **Low value** |
| --- | --- | --- |
| Archie beagle | 12 | 9 |
| Archie labradoodle | 0 | 0 |
| Buster | 21 | 2 |
| Charlie | 37 | 8 |
| George | 38 | 0 |
| Hope | 30 | 3 |
| Jess | 23 | 5 |
| Juno | 34 | 16 |
| Jupiter | 15 | 7 |
| Kess | 25 | 4 |
| Penny | 12 | 6 |
| Pepper | 17 | 10 |
| Poppy | 20 | 16 |
| Rue | 26 | 6 |
| Toby | 33 | 12 |
| Tutts | 38 | 5 |
| Zip | 30 | 10 |
| Zoe | 23 | 18 |
| ***Mean*** | ***24.11*** | ***7.61*** |
| ***SEM*** | ***2.33*** | ***1.19*** |

**Table S4.** Latency to engage with the first board during the unshifted condition with calculated mean, standard error of the mean, and upper and lower 95% confidence intervals.

| **Dog** | **T1** | **T2** | **T3** | **T4** | **T5** |
| --- | --- | --- | --- | --- | --- |
| Archie beagle | 1.61 | 1.16 | 2.84 | 3.18 | 1.75 |
| Archie labradoodle | 1.43 | 2.08 | 3.55 | 2.65 | 10.55 |
| Charlie | 13.88 | 35.71 | 7.55 | 77.3 | 120 |
| George | 3.06 | 1.85 | 2.61 | 1.01 | 1.26 |
| Hope | 0.83 | 1.5 | 1.13 | 1.28 | 1.26 |
| Jess | 0.87 | 1.5 | 0.93 | 0.88 | 1.19 |
| Kess | 1.35 | 1.05 | 1.03 | 1.06 | 1.01 |
| Penny | 1.63 | 3.25 | 6.58 | 1.98 | 1.96 |
| Pepper | 0.85 | 1.51 | 1.18 | 2.96 | 1.76 |
| Poppy | 0.81 | 21.41 | 61.98 | 3.16 | 4.83 |
| Rue | 0.81 | 1.91 | 1.88 | 0.92 | 1.7 |
| Toby | 0.86 | 15.96 | 2.31 | 15.18 | 7.8 |
| Tutts | 1.76 | 3.56 | 1.43 | 1.45 | 1.23 |
| Zip | 1.13 | 1.21 | 1.46 | 0.81 | 0.85 |
| Zoe | 1.23 | 1.04 | 1.53 | 1.45 | 3.2 |
| Juno | 0.71 | 1.18 | 1.55 | 1.33 | 0.95 |
| Jupiter | 1.1 | 3.15 | 3.91 | 2.33 | 4.43 |
| Buster | 1.16 | 1.56 | 0.86 | 1.71 | 1.26 |
| ***Mean*** | ***1.95*** | ***5.59*** | ***5.80*** | ***6.70*** | ***9.28*** |
| ***SEM*** | ***0.71*** | ***2.20*** | ***3.33*** | ***4.22*** | ***6.54*** |
| ***CI-95%*** | ***0.44*** | ***0.94*** | ***-1.24*** | ***-2.21*** | ***-4.53*** |
| ***CI+95%*** | ***3.45*** | ***10.24*** | ***12.83*** | ***15.61*** | ***23.08*** |

**Table S5.** Latency to engage with the first board during the shifted condition with calculated mean, standard error of the mean, and upper and lower 95% confidence intervals.

| **Dog** | **T1** | **T2** | **T3** | **T4** | **T5** |
| --- | --- | --- | --- | --- | --- |
| Archie beagle | 1.41 | 1.64 | 1.06 | 2.16 | 0.96 |
| Archie labradoodle | 0.86 | 1.12 | 2.65 | 2.48 | 1.06 |
| Buster | 1.04 | 2.51 | 1.94 | 1.09 | 1.4 |
| Charlie | 1.15 | 1.13 | 1.18 | 2.46 | 1.83 |
| George | 0.73 | 1.01 | 1.06 | 1.19 | 1.98 |
| Hope | 1.23 | 1.15 | 1.06 | 1.78 | 1.12 |
| Jess | 0.86 | 0.7 | 0.61 | 0.56 | 0.49 |
| Juno | 1.18 | 1.68 | 12.08 | 7.96 | 2.33 |
| Jupiter | 1.1 | 3.15 | 3.91 | 2.33 | 4.43 |
| Kess | 0.9 | 1 | 0.81 | 0.88 | 1.19 |
| Penny | 1.41 | 1.19 | 1.08 | 1.2 | 1.95 |
| Pepper | 1.36 | 2.18 | 1.1 | 1.83 | 1.53 |
| Poppy | 1.61 | 1.13 | 1.31 | 5.21 | 1.21 |
| Rue | 1.03 | 1.93 | 1.79 | 1.01 | 1.02 |
| Toby | 0.89 | 1.01 | 1.95 | 1.15 | 1.98 |
| Tutts | 1.56 | 0.48 | 3 | 26.7 | 6 |
| Zip | 0.03 | 0.9 | 1.17 | 1.08 | 0.98 |
| Zoe | 2.38 | 3.1 | 4.38 | 2.43 | 3.53 |
| ***Mean*** | ***1.15*** | ***1.50*** | ***2.34*** | ***3.53*** | ***1.94*** |
| ***SEM*** | ***0.11*** | ***0.18*** | ***0.63*** | ***1.43*** | ***0.33*** |
| ***CI-95%*** | ***0.92*** | ***1.11*** | ***1.02*** | ***0.52*** | ***1.25*** |
| ***CI+95%*** | ***1.39*** | ***1.89*** | ***3.66*** | ***6.54*** | ***2.64*** |

**Table S6.** Total time engaged with boards during the unshifted condition with calculated mean, standard error of the mean, and upper and lower 95% confidence intervals.

| **Dog** | **T1** | **T2** | **T3** | **T4** | **T5** |
| --- | --- | --- | --- | --- | --- |
| Archie beagle | 104.78 | 99.84 | 101.64 | 106.42 | 99.1 |
| Archie labradoodle | 63.01 | 62.35 | 112.36 | 48.15 | 100.95 |
| Buster | 100.55 | 85.53 | 103.03 | 107.33 | 99.1 |
| Charlie | 44.63 | 63.78 | 22.08 | 2.73 | 0 |
| George | 110.65 | 116.21 | 115.31 | 116.6 | 116.85 |
| Hope | 114.32 | 115.51 | 118.51 | 117.88 | 115.81 |
| Jess | 88.73 | 85.19 | 113.08 | 106.36 | 113.81 |
| Juno | 105.85 | 116.3 | 114.83 | 106.45 | 104.97 |
| Jupiter | 111.41 | 108.83 | 111.2 | 109.73 | 113.73 |
| Kess | 106.62 | 93.63 | 105.11 | 104.73 | 105.37 |
| Penny | 56.84 | 105.6 | 96.96 | 109.1 | 110.33 |
| Pepper | 99.7 | 106.1 | 109.8 | 108.48 | 99.78 |
| Poppy | 64.67 | 57.03 | 11.91 | 108 | 88.28 |
| Rue | 89.3 | 99.5 | 109.65 | 102.8 | 105.61 |
| Toby | 68.02 | 86.8 | 92.35 | 69.65 | 92.98 |
| Tutts | 9.85 | 100.41 | 113.88 | 108.2 | 112.16 |
| Zip | 107.66 | 112.61 | 113.63 | 113.82 | 113.99 |
| Zoe | 116.38 | 116.03 | 113.36 | 112.4 | 115.35 |
| ***Mean*** | ***86.83*** | ***96.18*** | ***98.82*** | ***97.71*** | ***100.45*** |
| ***SEM*** | ***5.42*** | ***4.67*** | ***7.74*** | ***7.40*** | ***6.67*** |
| ***CI-95%*** | ***72.21*** | ***86.62*** | ***83.61*** | ***83.19*** | ***87.31*** |
| ***CI+95%*** | ***101.45*** | ***105.75*** | ***114.03*** | ***112.24*** | ***113.59*** |

**Table S7.** Total time engaged with boards during the shifted condition with calculated mean, standard error of the mean, and upper and lower 95% confidence intervals.

| **Dog** | **T1** | **T2** | **T3** | **T4** | **T5** |
| --- | --- | --- | --- | --- | --- |
| Archie beagle | 86.01 | 104.92 | 113.18 | 86.75 | 97.03 |
| Archie labradoodle | 114.33 | 116.48 | 75.03 | 106.45 | 114.16 |
| Buster | 103 | 107.98 | 105.85 | 98.91 | 115.6 |
| Charlie | 114.18 | 113.73 | 67.48 | 108.55 | 15.31 |
| George | 113.56 | 111.51 | 117.33 | 111.65 | 114.56 |
| Hope | 114.48 | 118.43 | 109.73 | 114.83 | 116.23 |
| Jess | 115.7 | 114.66 | 108.45 | 111.86 | 113.9 |
| Juno | 109.53 | 113.81 | 108.69 | 114.41 | 118.95 |
| Jupiter | 107.81 | 115.53 | 109.88 | 112.6 | 116.83 |
| Kess | 98.45 | 101.05 | 87.18 | 97.83 | 106.86 |
| Penny | 104.15 | 113.87 | 106.71 | 111.98 | 114.18 |
| Pepper | 110.33 | 108.75 | 109.35 | 82.68 | 102.05 |
| Poppy | 97.41 | 101.48 | 53.58 | 24.45 | 113.83 |
| Rue | 110.78 | 107.8 | 98.05 | 111.38 | 111.97 |
| Toby | 100.32 | 108.64 | 101.38 | 115.71 | 114 |
| Tutts | 110.93 | 105.71 | 63.36 | 0.36 | 109.3 |
| Zip | 115.15 | 114.05 | 107.49 | 114.68 | 118.95 |
| Zoe | 115.32 | 118.26 | 113.01 | 116.3 | 118.78 |
| ***Mean*** | ***106.67*** | ***110.58*** | ***98.12*** | ***100.67*** | ***105.70*** |
| ***SEM*** | ***1.98*** | ***1.25*** | ***4.42*** | ***5.52*** | ***6.05*** |
| ***CI-95%*** | ***103.81*** | ***108.22*** | ***87.86*** | ***80.60*** | ***95.58*** |
| ***CI+95%*** | ***111.90*** | ***113.53*** | ***107.22*** | ***112.88*** | ***119.14*** |

**Table S8.** Number of food pieces consumed during the unshifted condition with calculated mean, standard error of the mean, and upper and lower 95% confidence intervals.

| **Dog Name** | **T1** | **T2** | **T3** | **T4** | **T5** |
| --- | --- | --- | --- | --- | --- |
| Archie beagle | 32 | 30 | 29 | 29 | 26 |
| Archie labradoodle | 9 | 10 | 14 | 12 | 24 |
| Buster | 40 | 32 | 36 | 31 | 34 |
| Charlie | 8 | 13 | 3 | 1 | 0 |
| George | 26 | 21 | 20 | 42 | 36 |
| Hope | 19 | 22 | 19 | 21 | 32 |
| Jess | 44 | 46 | 48 | 54 | 52 |
| Juno | 30 | 30 | 29 | 31 | 36 |
| Jupiter | 26 | 25 | 19 | 22 | 19 |
| Kess | 23 | 27 | 26 | 25 | 29 |
| Penny | 8 | 19 | 21 | 30 | 32 |
| Pepper | 25 | 27 | 24 | 27 | 27 |
| Poppy | 28 | 27 | 6 | 26 | 18 |
| Rue | 31 | 44 | 44 | 48 | 44 |
| Toby | 19 | 20 | 21 | 15 | 18 |
| Tutts | 1 | 27 | 30 | 37 | 33 |
| Zip | 26 | 40 | 47 | 36 | 39 |
| Zoe | 22 | 22 | 19 | 30 | 18 |
| ***Mean*** | ***23.17*** | ***26.78*** | ***25.28*** | ***28.72*** | ***28.72*** |
| ***SEM*** | ***2.65*** | ***2.25*** | ***2.96*** | ***2.96*** | ***2.77*** |
| ***CI-95%*** | ***17.58*** | ***22.03*** | ***19.03*** | ***22.47*** | ***22.87*** |
| ***CI+95%*** | ***28.75*** | ***31.52*** | ***31.53*** | ***34.97*** | ***34.57*** |

**Table S9.** Number of food pieces consumed during the shifted condition with calculated mean, standard error of the mean, and upper and lower 95% confidence intervals.

| **Dog Name** | **T1** | **T2** | **T3** | **T4** | **T5** |
| --- | --- | --- | --- | --- | --- |
| Archie beagle | 21 | 26 | 25 | 27 | 28 |
| Archie labradoodle | 45 | 36 | 30 | 27 | 23 |
| Buster | 26 | 23 | 24 | 27 | 28 |
| Charlie | 23 | 24 | 19 | 24 | 30 |
| George | 36 | 31 | 25 | 27 | 27 |
| Hope | 40 | 36 | 33 | 26 | 29 |
| Jess | 37 | 38 | 42 | 42 | 47 |
| Juno | 9 | 22 | 21 | 23 | 26 |
| Jupiter | 10 | 11 | 12 | 12 | 16 |
| Kess | 29 | 29 | 29 | 26 | 30 |
| Penny | 54 | 56 | 52 | 52 | 59 |
| Pepper | 30 | 29 | 23 | 27 | 21 |
| Poppy | 41 | 32 | 24 | 11 | 37 |
| Rue | 22 | 33 | 28 | 38 | 32 |
| Toby | 13 | 13 | 21 | 16 | 12 |
| Tutts | 25 | 24 | 5 | 0 | 18 |
| Zip | 38 | 44 | 43 | 47 | 52 |
| Zoe | 29 | 32 | 27 | 26 | 30 |
| ***Mean*** | ***29.33*** | ***29.94*** | ***26.83*** | ***26.56*** | ***30.28*** |
| ***SEM*** | ***2.88*** | ***2.48*** | ***2.58*** | ***2.98*** | ***2.85*** |
| ***CI-95%*** | ***23.27*** | ***24.72*** | ***21.38*** | ***20.26*** | ***24.25*** |
| ***CI+95%*** | ***35.40*** | ***35.17*** | ***32.28*** | ***32.85*** | ***36.30*** |

**Table S10.** Frequency of switches between boards during the unshifted condition with calculated mean, standard error of the mean, and upper and lower 95% confidence intervals.

| **Dog** | **T1** | **T2** | **T3** | **T4** | **T5** |
| --- | --- | --- | --- | --- | --- |
| Archie beagle | 8 | 12 | 9 | 8 | 12 |
| Archie labradoodle | 4 | 2 | 2 | 2 | 3 |
| Buster | 7 | 10 | 9 | 7 | 6 |
| Charlie | 9 | 3 | 1 | 0 | 0 |
| George | 4 | 1 | 4 | 4 | 6 |
| Hope | 5 | 1 | 1 | 3 | 3 |
| Jess | 7 | 12 | 3 | 6 | 3 |
| Juno | 6 | 1 | 4 | 5 | 6 |
| Jupiter | 6 | 2 | 2 | 3 | 1 |
| Kess | 5 | 9 | 4 | 7 | 7 |
| Penny | 9 | 3 | 3 | 4 | 4 |
| Pepper | 8 | 6 | 7 | 7 | 9 |
| Poppy | 10 | 4 | 1 | 3 | 2 |
| Rue | 14 | 10 | 4 | 7 | 5 |
| Toby | 7 | 3 | 5 | 2 | 4 |
| Tutts | 4 | 7 | 2 | 5 | 7 |
| Zip | 6 | 2 | 3 | 4 | 2 |
| Zoe | 1 | 1 | 2 | 1 | 2 |
| ***Mean*** | ***6.67*** | ***4.94*** | ***3.67*** | ***4.33*** | ***4.56*** |
| ***SEM*** | ***0.68*** | ***0.94*** | ***0.58*** | ***0.55*** | ***0.71*** |
| ***CI-95%*** | ***5.24*** | ***2.95*** | ***2.44*** | ***3.18*** | ***3.06*** |
| ***CI+95%*** | ***8.09*** | ***6.94*** | ***4.90*** | ***5.49*** | ***6.05*** |

**Table S11.** Frequency of switches between boards during the shifted condition with calculated mean, standard error of the mean, and upper and lower 95% confidence intervals.

| **Dog** | **T1** | **T2** | **T3** | **T4** | **T5** |
| --- | --- | --- | --- | --- | --- |
| Archie beagle | 10 | 6 | 4 | 6 | 7 |
| Archie labradoodle | 3 | 4 | 13 | 4 | 1 |
| Buster | 7 | 5 | 6 | 9 | 2 |
| Charlie | 3 | 3 | 9 | 5 | 4 |
| George | 6 | 3 | 1 | 4 | 3 |
| Hope | 4 | 2 | 5 | 2 | 1 |
| Jess | 2 | 2 | 4 | 6 | 5 |
| Juno | 2 | 1 | 2 | 1 | 1 |
| Jupiter | 5 | 0 | 1 | 2 | 1 |
| Kess | 11 | 8 | 11 | 7 | 11 |
| Penny | 6 | 4 | 8 | 7 | 4 |
| Pepper | 3 | 7 | 2 | 6 | 3 |
| Poppy | 7 | 6 | 5 | 1 | 3 |
| Rue | 8 | 8 | 14 | 8 | 4 |
| Toby | 8 | 4 | 6 | 2 | 0 |
| Tutts | 3 | 4 | 11 | 0 | 1 |
| Zip | 2 | 2 | 6 | 7 | 2 |
| Zoe | 2 | 1 | 4 | 1 | 2 |
| ***Mean*** | ***Mean*** | ***5.11*** | ***3.89*** | ***6.22*** | ***4.33*** |
| ***SEM*** | ***SEM*** | ***0.68*** | ***0.57*** | ***0.94*** | ***0.66*** |
| ***CI-95%*** | ***CI-95%*** | ***3.68*** | ***2.70*** | ***4.24*** | ***2.94*** |
| ***CI+95%*** | ***CI+95%*** | ***6.55*** | ***5.08*** | ***8.21*** | ***5.73*** |

**Table S12.** SNC Score calculation. The mean number of switches observed during the post-shift trials (trials 3-4) of the unshifted condition was subtracted from the number of switches of post-shift trials of the shifted condition.

| **Dog** | **Mean switches post-shift unshifted** | | **Mean switches post-shift shifted** | **SNC Score** |
| --- | --- | --- | --- | --- |
| Archie beagle | 8.5 |  | 5 | -3.5 |
| Archie labradoodle | 2 |  | 8.5 | 6.5 |
| Buster | 8 |  | 7.5 | -0.5 |
| Charlie | 0.5 |  | 7 | 6.5 |
| George | 4 |  | 2.5 | -1.5 |
| Hope | 2 |  | 3.5 | 1.5 |
| Jess | 4.5 |  | 5 | 0.5 |
| Juno | 4.5 |  | 1.5 | -3 |
| Jupiter | 2.5 |  | 1.5 | -1 |
| Kess | 5.5 |  | 9 | 3.5 |
| Penny | 3.5 |  | 7.5 | 4 |
| Pepper | 7 |  | 4 | -3 |
| Poppy | 2 |  | 3 | 1 |
| Rue | 5.5 |  | 11 | 5.5 |
| Toby | 3.5 |  | 4 | 0.5 |
| Tutts | 3.5 |  | 5.5 | 2 |
| Zip | 3.5 |  | 6.5 | 3 |
| Zoe | 1.5 |  | 2.5 | 1 |
